# Supplemental feeding during pregnancy compared with maternal supplementation during lactation does not affect schooling and cognitive development through late adolescence^1^,^2^,^3^

**DOI:** 10.3945/ajcn.113.063404

**Published:** 2013-10-16

**Authors:** Harold Alderman, Sophie Hawkesworth, Mattias Lundberg, Afia Tasneem, Henry Mark, Sophie E Moore

**Affiliations:** 1 From the Human Development Network, World Bank, Washington, DC (HA, ML, and AT); the International Food Policy Research Institute, Washington, DC (HA); the Medical Research Council (MRC), International Nutrition Group, London School of Hygiene and Tropical Medicine, London, United Kingdom; and the MRC Keneba, MRC Unit, Fajara, The Gambia (SH, HM, and SEM).

## Abstract

**Background:** The long-term impact of early malnutrition on human capital outcomes remains unclear, and existing evidence has come largely from observational studies.

**Objective:** We compared the impact of a nutritional supplement given during pregnancy or lactation in rural Gambia on educational performance and cognitive ability in offspring at their maturity.

**Design:** This study was a follow-up of a randomized trial of prenatal high protein and energy supplementation conducted between 1989 and 1994. Subjects were 16–22 y of age at follow-up, and information was collected on schooling achievement and cognitive ability by using the Raven's progressive matrices test, Mill Hill vocabulary test, and forward and backward digit-span tests.

**Results:** A total of 1459 individuals were traced and interviewed and represented 71% of the original cohort and 81% of the surviving cohort. There was no difference in cognitive ability or educational attainment between treatment groups by using any of the methods of assessment.

**Conclusion:** We have shown little evidence to support a long-term effect of prenatal protein-energy supplementation compared with supplementation during lactation on cognitive development in rural Gambians. This trial was registered at http://www.controlled-trials.com as ISRCTN72582014.

See corresponding article on page 1.

## INTRODUCTION

A primary challenge for nutrition policy in low-income settings is to position nutrition as an investment. Various models have produced estimates of the economic benefits from reducing malnutrition. These benefits stem from a combination of reductions in mortality and morbidity and increases in productivity over the lifetime of survivors (1, 2). However, with a few notable exceptions, the evidence for the contribution of nutrition to economic productivity has been based on indirect inferences, albeit with a fair consistency and regularity of results. There is, for example, extensive evidence that nutrition (both intrauterine growth restriction and stunting) affects cognitive capacity of children (3) and little doubt that cognitive (and noncognitive) ability contributes to school performance. In addition, economists have regularly explored how wages respond to both years of school and learning per year. Opportunities to follow these links for the same individual are rare.

Data from a randomized trial in Guatemala provided some support for the impact of early nutrition on earnings (4, 5). The study followed individuals who had been born in rural villages and randomly assigned to a community-based nutritional intervention in the 1960s. All pregnant women and children in 4 villages were eligible to receive a nutritional supplement drink as follows: 2 villages received the high-energy/high-protein Atole supplement, whereas the remaining 2 villages received the Fresco drink, which contained no protein and only one third of the energy (6). Male subjects who had received the high-energy/high-protein supplements before the age of 3 y earned, on average, 44% higher wages by the time they were between 25 and 42 y old. This group also had more schooling and higher cognitive test scores. As useful as this sample is, it has limitations. For example, the small number of villages did not allow for the cluster design to be accounted for in the analysis. In addition, although the intervention provided supplements to pregnant women and young children, the sample did not allow for the analysis of either intervention separately from the other. A recent systematic review of prenatal single- and multiple-micronutrient supplementation on offspring mental development showed little evidence to support a long-term effect, except some evidence to support n−3 fatty acids or multimicronutrients that had some positive effect (7). However, the evidence base for long-term effects of macronutrient supplementation remains weak, and thus, there are potential gains from the replication and expansion of these examples.

The current study addressed these tasks by returning to a cohort of children whose mothers were provided nutritional supplements in a randomized trial in the West Kiang region of The Gambia between 1989 and 1994 (8). With the use of a cluster randomized design, all pregnant women in 28 villages were assigned to a group who received either 2 protein-energy–fortified biscuits/d from 20 wk of gestation to delivery or a control group who received the biscuits for 20 wk postpartum. There was no group that received no intervention. The 2 biscuits provided ∼4250 kJ (1015 kcal) energy and 22 g protein/d and were made from local ingredients and prepared locally. This trial was registered at http://www.controlled-trials.com as ISRCTN72582014.

The mean birth weight in the intervention group was 136 g higher than in the control group, with a larger difference of 201 g observed in the nutritionally poor hungry season, relative to a mean 40-wk birth weight of ∼2850 g in the control group. The nutrition supplementation reduced the probability of having a low-birth weight baby from 17% to 11% (OR: 0.61) for all births and reduced risk of neonatal morality with an OR of 0.57 (8).

The cohort of this randomized study has been tracked over time to study changes in the physiology of children (9, 10). A total of 1317 of the children from the original cohort were revisited between November 2005 and August 2006 when aged between 11 and 17 y. No differences were shown in the physiology between the treatment and control populations in terms of body size, composition, blood pressure, and metabolic markers (11). In the current study, we retracked this cohort to assess human capital outcomes, including education and cognitive ability in children born to women in the original intervention compared with control groups.

## SUBJECTS AND METHODS

### Study population

The current study tracked all individuals who were born during the original study, whether or not they were interviewed in 2005 and 2006, including subjects who had moved out of the study area, primarily to the coast. The tracking of subjects and families began in February 2011, and fieldwork began in May 2011; data collection was completed in March 2012. The questionnaire was developed at the research site in West Kiang [Medical Research Council (MRC) Keneba] and field-tested and revised simultaneously with the training of interview teams. Approval for the study was granted by the joint Gambian Government/MRC Unit The Gambia Ethics Committee (project SCC1121v2), and all study participants gave informed written consent before participating in the study.

### Data collection

Data were collected during one-to-one interviews at the homes of participants and in a quiet location. A total of 6 interviewers were extensively trained at the start of the study period and conducted all interviews during the year of follow-up; interview techniques were regularly checked for standardization by the field supervisor. All data-collection staff were blinded to the original treatment allocation of participants.

#### Data on education

Extensive questions during the interview were used to assess a number of factors related to the schooling achievement of participants including the number of years spent at school (in both Arabic and English schooling systems and taking into account missed or repeated years) and the highest grade achieved as well as whether the individual was still in school (English medium schools: primary school grades 1–6, middle school grades 7–9, and secondary school grades 10–12; Arabic schools: grades 1–12).

#### Data on cognitive assessment

Because the sample ranged between 16 and 22 y of age at the time of follow-up, and English fluency may have varied on the basis of the level of education of participants and their parents, Raven's progressive matrices (Pearson Publishing Company) were used as the primary instrument to assess cognitive ability. Raven's progressive matrices are a nonverbal test of reasoning ability on the basis of figural stimuli that measure the ability to form comparisons, reason by analogy, and organize spatial perceptions into systematically related wholes. In addition, the team collected the following 2 other measures of cognitive functioning: the Mill Hill vocabulary test (translated to Mandinka and Fulani) and the backward and forward digit-span test. The Mill Hill test was used to ask respondents to define a series of words in increasing difficulty, in this case in multiple-choice framework. In the digit-span test, respondents were presented with a series of numbers and asked to repeat them immediately. In the backward digit-span test, respondents were asked to repeat the numbers in reverse order (12).

The cognitive tests (Raven's, Mill Hill, and digit span) are only internally comparable (ie, they can be used to make comparisons between groups within the sample but should not be used to make comparisons between the sample and other samples). In principle, the Raven's test consists of 5 separate sections of 12 questions each. In field tests, it was determined that the use of the first 3 Raven's sections yielded a sufficient distribution to permit comparisons.

#### Additional measurements

Anthropometric measurements were also conducted during the interview with study participants. Height was measured by using a daily calibrated stadiometer (Seca Leicester stadiometer; Chasmors Ltd) to the nearest 0.1 cm, and weight was measured to the nearest 0.1 kg by using digital scales (Tanita digital scales; Chasmors Ltd). Finally, individuals were asked about any salaried or other work that they were currently undertaking and wages accrued for these jobs.

### Statistical analysis

All statistical analyses were performed with Stata 12.1 software (Stata Corp). Comparisons between group means were made by using 2-tailed *t* tests. Standard multivariate tests were performed to verify that attrition was not correlated with the treatment (13, 14). Main results were derived by using ordinary least-squares (OLS) regression allowing for clustering within each of the study villages. Although some of the dependent variables were ordered or categorical rather than strictly continuous, other regression techniques, such as ordered probit, yielded similar results (data not presented). The association between maternal supplementation and offspring cognitive performance and school achievement was assessed by intention-to-treat analysis. Statistical tests of the significance of the intervention on outcomes were conducted twice as follows: first, as a simple comparison of means between the treatment and control groups, and second, with the addition of a set of other variables to eliminate any differences in observed outcomes that might have been due to other factors, independently of the treatment. Even in randomized trials, such adjusted results can have reduced SEs.

Both paternal education and maternal education were included in regression analyses because parental education can influence education choices as well as the stimulation within the household that, in turn, influences cognitive development (3). These are, to a degree, also proxy variables for household wealth and family background as is maternal height. Moreover, maternal height as well as gestational age can have a bearing on the achieved height of the individual later in life and, thus, were additionally included in adjusted regressions. The language score and digit-span models include a dummy variable defined as one for individuals from households in which Mandinka was spoken as a primary language.

We allowed SEs in regressions to be clustered at the village level. The usual assumption is that errors in the regression variable estimates are independently and identically distributed across all observations. This assumption is violated if there are systematic differences in the outcome across villages, leading to correlation in the errors within villages, as well. For example, villages may differ systematically in household wealth, agricultural productivity, or the distance to markets; these factors vary across villages but are correlated across households within villages. If this is the case, the variable estimate (the point estimate of the impact of the treatment on the outcome) will still be unbiased, but estimated SEs may be too small, and lead to an incorrect inference, especially an improper rejection of the null hypothesis in favor of the alternative (type I error). In general, the power of the sample falls as the intragroup correlation rises because each individual provides less independent information. In this study, the within-village correlations were relatively low for all outcomes of interest (ranging from 0.02 to 0.07;* see* Supplementary Material Table 1 under “Supplemental data” in the online issue).

## RESULTS

**FIGURE 1.**
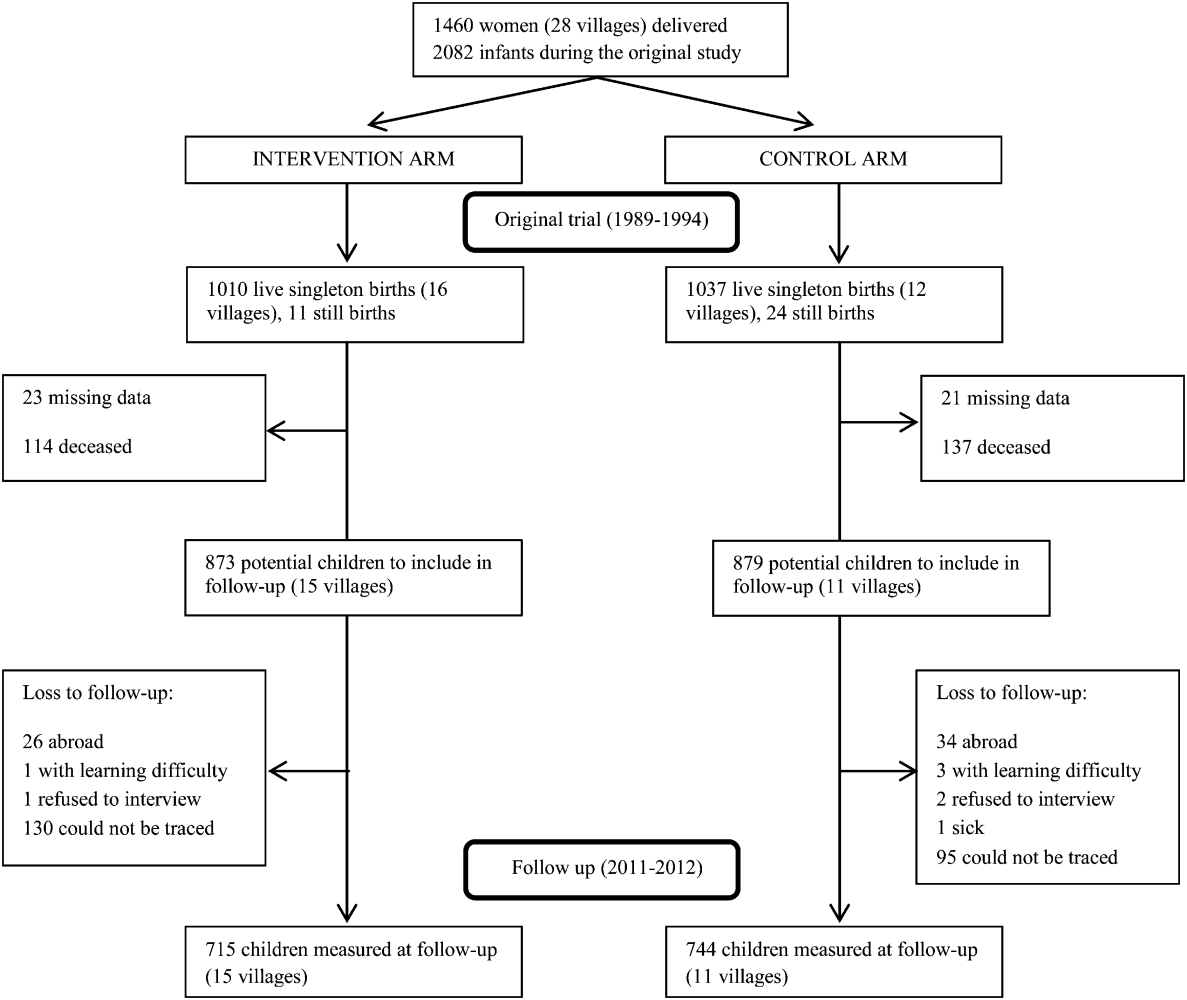
Flow of participants from original trial to current follow-up (2011–2012).

Of the 2047 live births in the original trial, 251 individuals were deceased, 60 individuals were out of the country, 8 individuals could not be interviewed, and 269 individuals were either missing before the 2005–2006 round (44 individuals) or untraceable in 2011–2012 (225 individuals) (**Figure 1**). Thus, a follow-up sample of 1459 children remained, which corresponded to an attrition rate of 29%; as a share of the surviving sample, attrition was 19%. The entire sample from 2 villages, one from the control (7 individuals) and one from the treatment (2 individuals), were deceased, and thus, the number of villages declined between rounds. Of the 1459 children in the follow-up sample, one child with a birth weight of 740 g was dropped from the data used for the analysis. Descriptive statistics from the baseline of individuals lost to follow-up and those who remained in the sample are shown in **Table 1**. The only difference in measured characteristics between subjects who were recruited and subjects who could not be included was a marginal (2-mo) difference in age of participants. The OR for the intervention cohort of the probability of being lost to follow-up was 1.03 (95% CI: 0.79, 1.36) in a regression that included sex, year and season of birth, gestational age as measured by the Parkin score, and mother's height and parity. We tested whether the subsample that was not included in the follow-up study was different from the main sample with respect to determinants of birth weight by including a variable that accounted for the fact that the individual would be subsequently lost to follow-up in a regression of birth weight that also included the same covariates in the original study. This variable was not significant (*P* = 0.89). This result implied that the subsample that was not included in the follow-up study was not different from the main sample with respect to determinants of birth weight.

**TABLE 1:** Original trial characteristics for recruited individuals compared with subjects lost to follow-up in the current study

	Loss to follow-up*^1^*		Recruited		
	*n*	Mean ± SD	*n*	Mean ± SD	*^2^*
Age at start of follow-up (y)*^3^*	544	19.2 ± 1.5	1459	19.0 ± 1.5	<0.001*^4^*
F (%)	544	46.1 ± 49.9	1459	49.9 ± 50.0	0.142
Maternal weight (kg*)*	544	53.6 ± 7.4	1456	52.9 ± 6.9	0.138
Maternal height (m)	412	159.3 ± 6.2	1172	159.0 ± 6.1	0.441
Maternal parity	504	4.3 ± 2.7	1390	4.4 ± 2.5	0.559
Gestational age (Parkin score)	457	8.9 ± 1.5	1279	8.8 ± 1.6	0.229
Birth weight (g)	544	2910.6 ± 433.6	1459	2902.3 ± 427.4	0.568
Birth length (cm)	462	49.4 ± 2.2	1292	49.3 ± 2.3	0.552
Month of birth*^5^*	544	6.8 ± 3.6	1459	6.7 ± 3.6	0.583

1 Data were missing on 44 children from the 2047 children born during the original trial.

2 Calculated by using 2-tailed *t* tests adjusted for clustering at the village level.

3 Start date of follow-up was 4 February 2011.

4 Significant.

5 Represented by numbers 1–12 where 1 indicates January, 2 indicates February, etc, and ends with 12, which indicates December.

The mean age at follow-up was 19 y, and 50.1% of the recruited sample were boys. 42.2% of participants were still at school, and 35.7% of participants still resided in the rural West Kiang region where they were born. A total of 13.7% of participants’ mothers and 19.4% of participants’ fathers had received some form of formal education. Descriptive statistics of main-outcome variables of interest in the current survey according to randomly assigned group and sex are shown in **Table 2**. The mean birth weight was 2903 g and was 91 g higher for boys than girls (95% CI: 33.3, 149.0 g; *P* = 0.003) and 95 g higher for treatment children than control subjects (95% CI: −2.1, 191.3 g; *P* = 0.055). In the current round of data collection, boys were taller than girls, but there was no apparent difference in height between treatment and control samples. School initiation was virtually universal, whereby 94.6% of the recruited sample had been to school, and the mean highest grade attained was grade 7. There was no difference in grade attainment between boys and girls (mean difference: 0.2 grades; 95% CI: −0.2, 0.7 grades; *P* = 0.312). The difference in the highest grade attained between treatment and control groups was small and not significant (mean difference: 0.5 grades; 95% CI: −0.2, 1.2 grades; *P* = 0.167).

**TABLE 2:** Descriptive statistics at the 2011–2012 follow-up*^1^*

	Intervention					Control				
	M		F			M		F		
	*n*	Mean ± SD	*n*	Mean ± SD	*P*	*n*	Mean ± SD	*n*	Mean ± SD	*P*
Age (y)	368	19.6 ± 1.5	347	19.6 ± 1.5	0.906	363	19.5 ± 1.5	380	19.5 ± 1.6	0.710
Resident in West Kiang (%)	339	47.8 ± 50.0	324	48.5 ± 50.0	0.838	307	44.0 ± 49.7	331	55.0 ± 49.8	0.120
Currently in school (%)	341	48.4 ± 50.0	334	38.9 ± 48.8	0.059	343	46.4 ± 49.9	359	35.4 ± 47.8	0.008*^2^*
Mother received formal education indicator (%)	368	14.9 ± 35.7	347	13.3 ± 33.9	0.533	363	12.1 ± 32.7	380	14.5 ± 35.2	0.459
Father received formal education indicator (%)	368	18.2 ± 38.6	347	20.2 ± 40.2	0.537	363	20.1 ± 40.1	380	19.2 ± 39.4	0.880
Birth weight (g)	368	2997.1 ± 438.2	347	2904.2 ± 395.6	0.001*^2^*	363	2900.8 ± 431.4	380	2816.0 ± 408.5	0.121
Height (cm)	365	170.6 ± 7.7	344	160.2 ± 5.6	<0.001*^2^*	359	170.6 ± 8.5	377	161.2 ± 6.5	<0.001*^2^*
Any schooling (%)	368	92.7 ± 26.1	346	96.5 ± 18.3	0.161	361	95.0 ± 21.8	380	94.5 ± 22.9	0.725
Highest grade attained	368	7.2 ± 3.2	345	7.0 ± 2.8	0.217	361	7.7 ± 3.0	380	7.4 ± 3.2	0.550
Digit span (forward)	368	11.0 ± 2.9	345	10.4 ± 3.0	0.017*^2^*	361	11.3 ± 2.7	379	10.6 ± 2.8	0.038*^2^*
Digit span (backward)	368	5.2 ± 2.1	345	4.8 ± 1.9	<0.001*^2^*	360	5.5 ± 2.0	379	4.9 ± 2.3	0.042*^2^*
Raven's test	367	15.9 ± 5.2	344	13.4 ± 3.9	<0.001*^2^*	360	15.5 ± 5.4	379	13.5 ± 4.1	0.013*^2^*
Mill Hill vocabulary test	368	15.4 ± 2.8	344	14.4 ± 3.0	<0.001*^2^*	361	15.3 ± 3.0	378	14.4 ± 3.0	0.023*^2^*

1 Birth-weight data are from the original trial conducted between 1989 and 1994; all other data are from the follow-up study in 2011–2012. *P* values were calculated by using 2-tailed *t* tests for comparison of M and F and adjusted for clustering at the village level.

2 Significant (*P* < 0.05).

**FIGURE 2.**
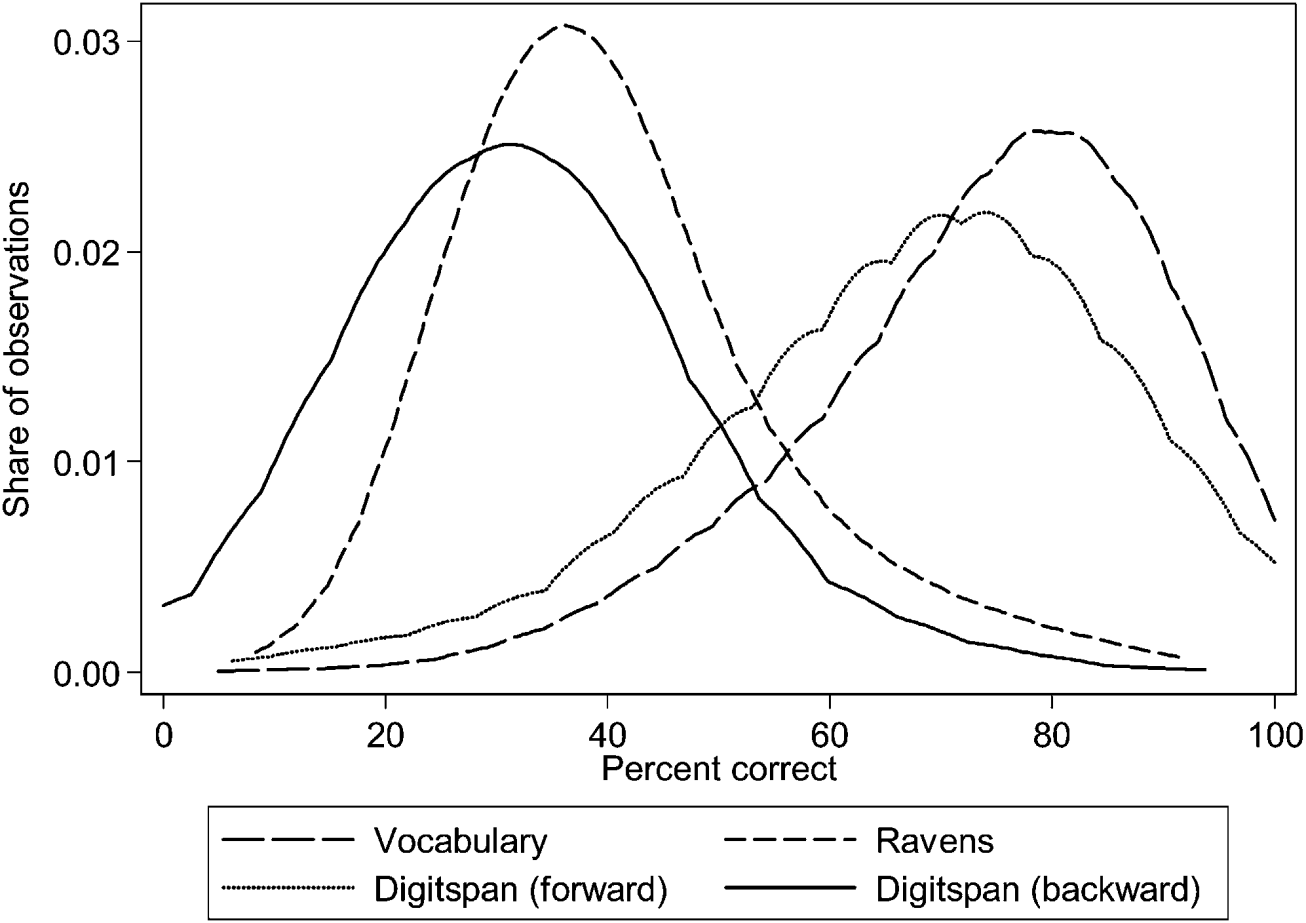
Distribution of cognitive test scores (percentages). Data presented are for all subjects at follow-up with groups combined.

Distributions of cognitive test scores for the different tests are shown in **Figure 2**. As illustrated, there was quite a bit of separation within each of the different cognitive tests. The median (range) percentages of correct answers were as follows: Raven's test: 36% (8–92%); forward digit span: 69% (6–100%); backward digit span: 31% (0–94%); and vocabulary test: 75% (5–100%).

Unadjusted regression results that tested whether the means differed across treatment and control groups are shown in **Table 3**. No impact of the intervention was observed on school attainment or any of the cognitive tests studied. However, because these differences may have been masked by other sources of influence, we also present results of adjusted OLS regressions that included a set of covariates in **Table 4**. All models shown in Table 4 were adjusted for age, sex, maternal height, maternal parity, gestational age by using Parkin score and season of birth. It was important to include the age of the individual in these models because the study population ranged in age from 16 y 4 mo to 22 y 7 mo. That was a sufficiently large range to include some young people who might still be growing or still be in school, which raised the possibility of a truncation in the data; because 285 people were <18 y old, this possibility was apparent. However, age was not correlated with treatment by design.

**TABLE 3:** Effect of maternal nutritional intervention on offspring education and cognitive assessment (unadjusted)*^1^*

	*n*	Unadjusted difference*^2^*	*P*
Height (cm)	1445	−0.21 (−1.57, 1.14)	0.747
Highest grade attained	1454	−0.49 (−1.20, 0.22)	0.167
Raven's test score	1450	0.21 (−0.58, 0.99)	0.593
Vocabulary test score	1451	0.01 (−0.59, 0.61)	0.979
Digit-span (forward) score	1453	−0.16 (−0.62, 0.29)	0.468
Digit-span (backward) score	1452	−0.18 (−0.51, 0.14)	0.250

1 *P* values were obtained from an intention-to-treat analysis by using ordinary least-squares regression. SEs were adjusted for clustering at the village level.

2 All values are regression coefficients; 95% CIs in parentheses.

**TABLE 4:** Regression results with covariates*^1^*

	Height (cm)	Highest grade attained	Raven's test score	Vocabulary test score	Digit-span (forward) score	Digit-span (backward) score
Village intervention allocation	−0.423 (−1.589, 0.743)	−0.503 (−1.180, 0.174)	0.163 (−0.595, 0.921)	−0.072 (−0.651, 0.507)	−0.187 (−0.605, 0.231)	−0.132 (−0.410, 0.147)
Mandinka as main language indicator	—	—	—	0.775 (0.141, 1.408)*	0.334 (−0.287, 0.955)	−0.492 (−0.847, −0.137)**
Born in the hungry season	−0.406 (−0.973, 0.162)	−0.043 (−0.364, 0.278)	0.015 (−0.665, 0.695)	−0.073 (−0.474, 0.328)	−0.231 (−0.523, 0.060)	0.062 (−0.108, 0.232)
F	−9.752 (−10.685, −8.820)**	−0.235 (−0.736, 0.266)	−2.195 (−3.014, −1.377)**	−0.936 (−1.348, −0.523)**	−0.688 (−1.070, −0.307)**	−0.514 (−0.811, −0.217)**
Maternal parity at baseline	−0.071 (−0.190, 0.050)	−0.042 (−0.119, 0.035)	−0.082 (−0.167, 0.002)^†^	−0.014 (−0.074, 0.046)	0.017 (−0.037, 0.070)	0.015 (−0.040, 0.070)
Indicator for missing maternal parity	0.073 (−2.410, 2.555)	0.569 (−0.474, 1.613)	0.980 (0.055, 1.904)*	0.505 (−0.367, 1.377)	−0.247 (−1.131, 0.637)	0.210 (−0.387, 0.808)
Gestational age (Parkin score)	0.548 (0.302, 0.793)**	0.147 (−0.014, 0.308)^†^	0.260 (0.108, 0.411)**	0.116 (0.027, 0.205)*	0.144 (0.055, 0.232)**	0.079 (−0.013, 0.171)^†^
Maternal height at baseline (20 wk gestation) (cm)	0.317 (0.242, 0.392)**	0.010 (−0.024, 0.043)	0.062 (0.005, 0.120)*	0.023 (−0.015, 0.060)	0.007 (−0.024, 0.039)	0.007 (−0.009, 0.023)
Indicator for missing maternal height	49.883 (38.525, 61.241)**	1.198 (−4.260, 6.655)	10.275 (0.984, 19.567)*	3.589 (−2.469, 9.647)	1.399 (−3.546, 6.345)	1.059 (−1.551, 3.669)
Father received formal education indicator (yes or no)	0.748 (−0.300, 1.796)	0.253 (−0.214, 0.719)	0.128 (−0.757, 1.012)	0.639 (0.237, 1.042)**	0.758 (0.403, 1.113)**	1.038 (0.810, 1.267)**
Mother received formal education indicator (yes or no)	−0.434 (−1.637, 0.768)	−0.003 (−0.564, 0.558)	−0.154 (−0.761, 0.453)	−0.342 (−0.683, −0.000)*	−0.135 (−0.496, 0.227)	−0.340 (−0.664, −0.017)*
Current age	0.546 (0.282, 0.810)**	0.308 (0.151, 0.466)**	0.289 (0.096, 0.482)**	0.097 (−0.024, 0.217)	−0.122 (−0.228, −0.016)*	−0.053 (−0.129, 0.023)
Constant	105.364 (91.746, 118.983)**	−0.998 (−8.251, 6.256)	−1.998 (−12.565, 8.568)	8.167 (1.738, 14.596)*	10.736 (5.479, 15.992)**	4.808 (1.834, 7.781)**
Observations	1445	1454	1450	1450	1450	1449
*R*^2^	0.387	0.044	0.081	0.045	0.037	0.062
Mean	165.6	7.3	14.6	14.9	10.8	5.1

1 All values are regression coefficients; 95% CI in parentheses. Values were determined from an intention-to-treat analysis using ordinary least-squares regression. SEs were adjusted for clustering at the village level. *P < 0.05, ** *P* < 0.01, ^†^*P* < 0.10.

As indicated in Tables 3 and 4, there seemed to be little distinction between subjects whose mothers received the treatment during pregnancy and subjects whose mothers received nutrition postpartum. There were a few points of secondary interest for this article that are shown in Table 4. In particular, both sex and age were significant across regressions in Table 4. The Parkin score and maternal height picked up some of the variance in outcome variables as well. However, there was not a pattern in outcomes that stemmed from the season of birth, although there was such a pattern in the original study of birth weight.

## DISCUSSION

This study showed no evidence that maternal protein-energy supplementation during pregnancy compared with supplementation during lactation affected offspring cognitive ability or school performance in rural Gambia. Despite marked benefits of supplementation during pregnancy on infant outcomes (infant size at birth and survival) in the original trial, no long-term effects on cognitive ability have been observed in the surviving cohort, whom we were able to trace and interview. There were 2 competing interpretations underlying this result, which we could not distinguish between. One possibility is that the differences in nutritional status that were achieved with supplementation did not contribute to a lasting benefit that could be measured in young adults. A second possibility is that the supplements provided to the control group postpartum generated positive benefits to control children strong enough to match the benefits of the treatment given to mothers who were pregnant. Because the initial trial was designed to assess the impact of supplements on birth weight rather than lifetime development, the provision of biscuits to the control group after the main outcome was measured was motivated by research ethics, and at the time, this provision was not believed to confound results. Moreover, the control group received iron and folate supplements during pregnancy as well as recommended antenatal care, the provision of which are consistent with research ethics but may have led to health benefits relative to the care generally received in rural Gambia.

The hypothesis that supplements to the control group provided a benefit similar to prenatal supplements might have been related to the toll that pregnancy, childbearing, and child-rearing take on mothers. However, there is no evidence that protein-energy supplementation of lactating women improved their breast-milk quality or quantity in The Gambia (15). This particular result was in keeping with one of the main conclusions of the classic Bacon Chow study (16), as well as more-recent trials of supplements to women affected by HIV/AIDS (17). Even in this case, the energy stores of the lactating mother could have been affected by the supplement. An exhausted mother will be less able and available to interact with her child patiently, provide stimulation, and form healthy and secure attachments (18). Such stimulation can counter risks associated with low birth weight (19). The postpartum receipt of nutrition supplementation in mothers in the control group seemed to eliminate any differences in the later cognitive development that might have resulted from prenatal supplementation.

The less optimistic possibility that the improvement in birth outcomes as measured by infant size and survival did not translate into a measureable impact at a later date has not been unprecedented; differences in early growth attributed to multiple micronutrient supplementation during pregnancy in Burkina Faso during pregnancy were shown to attenuate by age 30 mo (20). As the original study in The Gambia did not include a control group who received no intervention, a direct test of the impact of either prenatal supplements or those during lactation relative to the general population was not possible within the research design.

Given that inadequate maternal nutrition is a recognized risk factor in child development (3), the current study specifically tested the hypothesis that nutritional supplementation to mothers during pregnancy would have beneficial effects on the cognitive ability of their offspring as young adults. Given of the time frame from exposure to follow-up, this hypothesis was predicated on any prenatal effect being robust enough to persist despite competing exposures during infancy and childhood. In contrast with the Guatemalan trial (5), our study provided no supplements to children. Another distinction is that, in The Gambia, children generally received more schooling. In the Guatemalan sample, adults had only attended school for an average of 4 y. The mean highest grade attained within the Gambian cohort was grade 7, which indicated that, on average, this group of young adults had attended school for a minimum of 7 y. Therefore, it is possible that this increased exposure to schooling has swamped any prenatal effect.

Two additional explanations for the absence of a difference are possible but unlikely. First, differential survival could lead to an increase in the number of relatively weak children who remained in the treatment sample. However, the small difference in the overall mortality indicated in Figure 1 was not large enough to generate such an outcome. Second, it is possible that treatment children developed a skill or attribute that was in great demand in the marketplace but was not captured adequately by measures used in the current study. However, because of the absence of any differences in height or cognitive abilities, we believe that this interpretation is also unlikely. In addition, we were not able to look at wages because most of the sample were still at school at the point of follow-up, and hence, any impact on economic capacity was not possible. A limitation of the data collected was our inability to look at years at school and grade attained because of the post hoc realization of the complexity of the data that arose from the different schooling systems in The Gambia, with some children who attended both English medium and Koranic institutions.

One possibility is that the study was not powered to pick up differences in outcomes. Although post hoc power calculations did not provide additional information to that already present in the CIs, we, nevertheless, verified that the sample was adequate to confirm effect sizes of 0.2–0.3 (*see* Supplementary Material Table 1 under “Supplemental data” in the online issue) by using the observed SEs and intracluster correlations actually observed.

In conclusion, although protein-energy supplementation is a recommended intervention to improve birth outcomes, the current long-term follow-up does not provide evidence that it is more promising than a similar supplementation during lactation in terms of cognitive ability. Of relevance, this supplementation regimen did not result in differences in a number of other outcomes, including infant and childhood growth (9) and, in later childhood, blood pressure (10) and body composition (9). This study was not the first to find no sustained effect of a maternal intervention (pregnancy or lactation) on the cognitive development of offspring; a study conducted in Colombia over 3 decades ago also reported similar findings (21). Taken together, these results could indicate that the observed effects from the Guatemalan trial (4, 5) reflect the benefit of long-term supplementation to the infant postnatally or a combination of both prenatal and postnatal nutrition for cognitive ability. Data from a large cluster-randomized trial of counseling to exclusive breastfeeding showed that prolonged and exclusive breastfeeding improved cognitive development at 6.5 y of age (22). This result, together with the well-documented association between stunting in early childhood and poor cognitive development (23), and the importance of the early postnatal period for brain development (24) could suggest that improving nutrition during infancy may be of greater benefit for long-term economic gains than is supplementation during pregnancy alone.

## Supplementary Material

Supplemental data
