# Supplementary material for: Supplemental feeding during pregnancy compared with maternal supplementation during lactation does not affect schooling and cognitive development through late adolescence1
Source: Am J Clin Nutr. 2013 Oct 16;99(1):122–9. doi: 10.3945/ajcn.113.063404 (PMC3862451; doi:10.3945/ajcn.113.063404)
Supplement: Supplemental data [file supp_99_1_122__index.html]

Supplemental data 

# Supplemental feeding during pregnancy compared with maternal supplementation during lactation does not affect schooling and cognitive development through late adolescence

## Supplemental data

**Files in this Data Supplement:**

- Supplemental data - Table 1
