# Supplementary material for: Supplemental feeding during pregnancy compared with maternal supplementation during lactation does not affect schooling and cognitive development through late adolescence1
Source: Am J Clin Nutr. 2013 Oct 16;99(1):122–9. doi: 10.3945/ajcn.113.063404 (PMC3862451; doi:10.3945/ajcn.113.063404)
Supplement: Supplemental data [file 113.063404_ajcn063404SupplementaryData1.doc]

**Supplementary Material Table 1:** Post-hoc power calculations

|  | **n** | **J** | **mean** | **sd** | **Intra-cluster correlation** | **Minimum detectable effect size (standardised)** | **Minimum detectable effect size (level)** | **Observed difference (level)** |
| --- | --- | --- | --- | --- | --- | --- | --- | --- |
| Highest grade | 1454 | 26 | 7.32 | 3.06 | 0.07 | 0.30 | 0.92 | 0.49 |
| Height | 1445 | 26 | 165.65 | 8.73 | 0.02 | 0.21 | 1.83 | 0.21 |
| Vocabulary test | 1451 | 26 | 14.88 | 3.02 | 0.05 | 0.30 | 0.91 | 0.01 |
| Raven’s test | 1450 | 26 | 14.58 | 4.81 | 0.02 | 0.23 | 1.11 | 0.21 |
| Digitspan (forward) | 1453 | 26 | 10.82 | 2.89 | 0.02 | 0.23 | 0.67 | 0.16 |
| Digitspan (backward) | 1452 | 26 | 5.08 | 2.11 | 0.02 | 0.23 | 0.49 | 0.18 |
